# Virologic suppression and associated factors in HIV infected Ugandan female sex workers: a cross-sectional study

**DOI:** 10.4314/ahs.v21i2.15

**Published:** 2021-06

**Authors:** Darius Owachi, Godwin Anguzu, Joanita Kigozi, Janneke Cox, Barbara Castelnuovo, Fred Semitala, David Meya

**Affiliations:** 1 Department of Infectious Diseases, Kiruddu National Referral Hospital, Kampala, Uganda; 2 Department of Research, Infectious Diseases Institute, Makerere University, Kampala, Uganda; 3 Outreach Department, Infectious Diseases Institute, Makerere University, Kampala, Uganda; 4 Department of Infectious Diseases and Immunology, Jessa Hospital, Hasselt, Belgium; 5 Department of Medicine, Makerere University, Kampala, Uganda

## Abstract

**Introduction:**

Key populations have disproportionately higher HIV prevalence rates than the general population.

**Objective:**

To determine the level of virologic suppression and associated factors in female Commercial Sex Workers (CSW) who completed six months of ART and compare with the female general population (GP).

**Methods:**

Clinical records of CSW and GPs who initiated ART between December 2014 to December 2016 from seven urban clinics were analyzed to determine virologic suppression (viral load < 1000 copies/ml) and associated factors.

**Results:**

We identified 218 CSW and 182 female GPs. CSW had median age of 28 (IQR 25–31) vs 31 (IQR 26–37); median baseline CD4 446 (IQR 308–696) vs 352 (IQR 164–493) cells/microL; and optimal ART adherence levels at 70.6% vs 92.8% respectively, compared to GP. Virologic suppression in CSW and GPs was 85.7% and 89.6% respectively, P=0.28. Overall virologic suppression in CSW was 55% while Retention in care after 6 months of ART was 77.5%. Immediate ART initiation (<2weeks) and tuberculosis independently predicted virologic suppression in CSW with adjusted odds ratios 0.07 (95% C.I. 0.01–0.55, P=0.01) and 0.09 (95% C.I. 0.01–0.96, P=0.046) respectively.

**Conclusion:**

Virologic suppression in both groups is similar, however, intensified follow-up is needed to improve treatment outcomes

## Introduction

HIV positive key populations such as commercial sex workers (CSW) have disproportionately high HIV prevalence rates and contribute nearly half of the global new HIV infection rates^1^,^2^. In Uganda, the estimated HIV prevalence in CSW is 37.2%, higher than the national HIV prevalence estimates of the general population at 6.2%^3^,^4^. However, achieving virologic suppression by use of antiretroviral therapy (ART) reduces the risk of transmission of HIV by 98% in high-risk groups^5^–^9^.

The UNAIDS 90-90-90 targets: (i) to diagnose 90% of all HIV-positive persons, (ii) to initiate ART in 90% of those diagnosed, and (iii) to achieve viral suppression for 90% of those on ART; aim to reduce the number of new global HIV infections to below 500,000 by 2020^10^. However, ART coverage and virologic suppression in key populations remain significantly low despite the relatively high burden of HIV in these population groups. A study in Cameroon found that ART coverage in CSW was 13% compared to 56.5% for the HIV general population^11^. In Zimbabwe, a study involving CSW found a low level of overall virologic suppression at 49.5%^12^. Factors that could contribute to poor HIV treatment outcomes in key populations include stigma, discrimination, physical violence, abuse of human rights, criminalization and imprisonment^2^,^13^–^15^.

In this study, we determined the retention in care, HIV virologic suppression and the factors associated with virologic suppression in CSW and the female general HIV-positive population (GPs), who had initiated and completed six months (24 weeks) of ART between December 2014 and December 2016 in urban HIV clinic settings, in Uganda.

## Methods

### Study setting

This was a cross-sectional study conducted at seven urban public health centres within the capital Kampala, Uganda. With support from the Ugandan government, the Centers for Diseases Control (CDC) and the Infectious Diseases Institute (IDI), these health centres provide free HIV care services to nearly 40,000 people. Services offered include HIV testing and counselling, provision of ART, HIV prophylaxis, Antenatal and TB services, Laboratory Diagnostic services, psychosocial support counselling and community health camps.

Key Population (KP) specific health services provided include targeted community outreach testing, health education, condoms and lubricants, ART and psychosocial counselling services. KPs are identified and tested from communities with the aid of KP-peer network system and linked to a primary health centre where they are enrolled in HIV care. By 2014, all HIV positive KPs were immediately initiated on ART as guided by the WHO 2014 treatment guidelines^1^.

At the time, the general population (GP) were initiated on ART within 2 weeks or longer from the time of enrolment in care. Both KP and GP who initiated ART would be given monthly clinic appointments to monitor their adherence by pill count method (the proportion of the number of pills taken divided by the number of expected pills). Adherence would be categorized as good if > 95% of pills are taken, moderate if between 85 – 94%, and poor if below <85% of pills taken. In this study, adherence was measured as an average of the last six subsequent visits in the past six months of ART initiation.

After completion of six months of ART, the first viral load measurement to assess for virologic suppression. Viral load samples were collected by dried blood sample (DBS) method and processed using the Abbott RealTime HIV-1 assay for viral load measurement. The lowest level of detection of viral copies by the Abbott RealTime HIV-1 assay was 75 copies/ml. However, the national guidelines define HIV virologic suppression as a viral load threshold <1,000 viral copies/ml^16^,^17^. Other routine services provided at each clinic visit comprised screening for opportunistic infections, regular counselling anadherence support and laboratory diagnostic tests.

### Study Procedure

All medical records (both paper and electronic data) of the patients who enrolled and started ART between December 2014 until December 2016 were reviewed. CSWs were identified from the Key populations' register while GPs were identified from the Pre-ART register. CSW were selected by consecutive sampling method given their finite number in the clinics. GPs were selected by systematic sampling of every 70^th^ patient and on the basis of having viral load result until the sample size was attained.

We analyzed both paper copy and electronic clinical records of patients to obtain data on virologic outcome and factors associated with virologic suppression. At the health centres, clinical data is stored in both hard copies and on an electronic patient-level data system, the open-source Open-MRS (https://openmrs.org/). The clinical records are based on the WHO HIV care/ART card^18^.

Ethical approval for the study was obtained from the Makerere University institutional review board: School of Medicine Research Ethics Committee (SOMREC). A waiver of consent for the study was granted by SOMREC, on the basis that the extracted data were routinely collected at the health centres and the study did not involve direct interaction with patients. Data were de-identified and analyzed anonymously to maintain patient privacy. The study was also registered with the Uganda National Council of Science and Technology.

### Data collection and statistical analysis

Data were extracted using a standardized data collection tool and entered into EpiData version 3.1 software (The EpiData Association, Odense, Denmark) for data entry error checks before being exported to STATA version 14.0 (StataCorp, Texas, USA) for analysis.

Baseline characteristics of both CSWs and female GPs (with and without stratification according to the virologic outcome) were compared using Pearson's Chi-Square test in case of frequencies and Wilcoxon ranksum test in case of medians.

Viral load results were categorized as virologic suppression (HIV viral load <1,000copies/ml) and virologic non-suppression (HIV viral load ≥1000 copies/ml). Virologic suppression rates between CSW and GPs were compared using Pearson's Chi-Square test. Virologic status was also categorized as undetected viral load (Viral load <75copies/ml) and detected viral load (any viral load >75 copies/ml), and the rates compared using the Pearson's Chi-Square test.

Retention in care in CSW was categorized as active (when a patient attends all expected clinic visits in the past six months of ART or one who misses one visit but resumes the subsequent visits) or not-active (when a patient fails to attend more than two consecutive expected clinic visits in the past six months) and was expressed in proportions.

We used the logistic regression model to identify predictors of virologic suppression. Variables that had a threshold P-value < 0.2 at bivariate analysis and changed estimates by at least 10% when adjusted for, were considered in the multivariate analysis. In addition, other factors with higher p-values >0.2 but changed estimates by at least 10% when adjusted for, were also considered in the final model. Variables with missing proportions >10% (e.g. baseline haemoglobin and care entry point) in the CSW group were not considered in the multivariate analysis as they would bias the estimates of the model. Factors on the causal pathway for virologic suppression, such as adherence, were not adjusted for in the logistic regression model. Two-sided P-values of ≤ 0.05 level of significance were considered to be statistically significant.

## Results

### Baseline Characteristics

A total of 400 records of female patients were reviewed, of which 218 (54.5%) were commercial sex workers, (CSW) while 182 (45.5%) were female GPs. The baseline characteristics of CSWs and GPs are summarized in Table 1.

**Table 1:** Baseline Characteristics of study populations

Variable	Commercial Sex Workers N_1_ = 218 (%)	Female General Population N_2_ = 182 (%)	P-value
**Age**, median (IQR)	28 (25–31)	31 (26 –37)	<0.01^1^
**History of Tuberculosis**			
Yes	6 (2.9%)	8 (4.4%)	0.417
No	201 (97.1%)	172 (95.6%)	
**History of S.T.I.s**			
Yes	44 (21.8%)	6 (3.8%)	<0.01
No	158 (78.2%)	153 (96.2%)	
**Baseline CD4 cell count**, median (IQR)	446 (308 –696)	352 (164 –493)	<0.01^1^
**Baseline CD4 count, categorized**			
CD4 below 500 cells/µL	118 (59.6%)	140 (76.9%)	<0.01
CD4 above 500 cells/µL	80 (40.4%)	42 (23.1%)	
**Baseline Hemoglobin**, median (IQR),	12.3 (11.1 –13.2)	12 (11.3 –13.7)	0.95^1^
**Baseline ART Regimen**			
Zidovudine-based regimen	24 (11.9%)	56 (30.8%)	<0.01
Tenofovir-based regimen	177 (88.1%)	126 (69.2%)	
**Median time to ART Initiation** (weeks)	0 (0 –13)	4 (0 –8)	
**Time Duration from enrolment in care** **to start of ART**			
Immediate start (<2 weeks)	89 (44.5%)	54 (30.2%)	<0.01
Delayed start (≥3 weeks)	79 (39.5%)	99 (55.3%)	
Transfer-in while on ART	32 (16.0%)	26 (14.5%)	
**Adherence to ART**			
Good (>95% adherence)	127 (70.6%)	167 (92.8%)	<0.01
Fair (85–94% adherence)	23 (12.8%)	10 (5.5%)	
Poor (<84% adherence)	30 (16.6%)	3 (1.7%)	
**Care Entry Point**			
Out-Patient Department	81 (59.1%)	100 (74.6%)	
Community outreach	36 (26.3%)	1 (0.8%)	<0.01^2^
Antenatal clinic	3 (2.2%)	29 (21.6%)	
Transfer-in	16 (11.7%)	3 (2.2%)	
Tuberculosis clinic	1 (0.7%)	1 (0.8%)	

P - Pearson's Chi-Square P-value; P^1^ - Wilcoxon rank-sum test P-value; P^2^- Fishers exact P-value

Missing ; History of tuberculosis 13/400 (3.3%), History of S.T.I.s 39/400 (9.8%), Baseline CD4 cell count 20/400 (5.0%), Baseline hemoglobin 324/400 (81.0%), Baseline ART Regimen 17/400 (4.2%), time to ART Initiation 21/400 (5.3%), Adherence to ART 40/400 (10.0%), Care Entry Point 129/400 (32.3%).

The CSW had a younger median age of 28 years (IQR 25–31) compared the female GPs of median age 31 years (IQR 26–37). The CSW had a higher median baseline CD4 cell count of 446 cells/µL (IQR 308–696) compared to the median baseline CD4 cell count of the female GP at 352 cells/µL (IQR 164–493). The prevalence of sexually transmitted infections (STI) was higher in the CSW compared to the female GPs at 21.8% versus 3.8% respectively, P < 0.01. However, the prevalence of Tuberculosis was similar in both population groups; 2.9% in the CSW and 4.4% in the female GP, P = 0.417.

The median time to initiation of ART in the CSW was 0 weeks (IQR 0 - 13) with 44.5% starting ART within 2 weeks while 39.5% delayed ART initiation by 3 weeks and longer from enrolment in care. The median time to initiation of ART in GPs was 4 weeks (IQR 0 – 8) with 30.2% starting ART within 2 weeks from enrolment into care while 55.3% started ART beyond 3 weeks from the time of enrolment in care. Adherence to ART was significantly suboptimal in the CSW group with 70.6% achieving good adherence to ART versus 92.8% in the GPs, P <0.01.

### Retention in care for CSW

**Figure 2:**
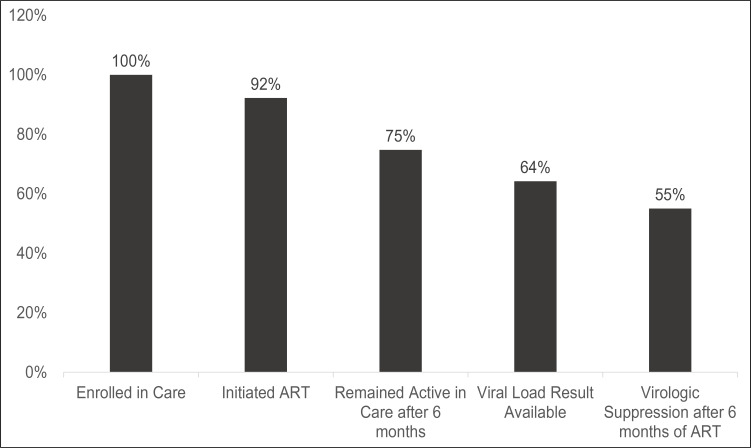
Care Cascade for Ugandan HIV positive Commercial Sex Workers on ART

Of the 218 CSWs enrolled in care, 169 (77.5%) had remained in care after 6 months of follow-up while 49 (22.5%) were lost to follow-up (Figure 2).

### Virologic Suppression

Of the 201 CSW who initiated ART, 140 KPs had a viral load done of which 120 (85.7%) achieved virologic suppression (Viral Load <1000 copies/ml). Of the 182 GPs who initiated ART and had a viral load done, 163 (89.6%) were virologically suppressed. Both population groups had similar levels of virologic suppression, P = 0.286. Assuming viraemia for CSW who did not have viral load result, the overall virologic suppression was 55% (Figure 2).

Undetected viral load (Viral load <75 copies/ml) was observed in 113 (80.7%) of the 140 CSW and in 138 (75.4%) of the 182 female GPs, P = 0.256). Assuming viraemia for those with unknown viral load result, the overall virologic suppression in CSW is 51.8%.

### Factors associated with Virologic Suppression

At bivariate analysis, factors associated with virologic suppression in CSW included a history of Tuberculosis, duration between enrolment in care to initiation of ART and care entry point (Table 2). In comparison, no variables were associated with virologic suppression in GPs (Table 3).

**Table 2:** Baseline Characteristics of Female Commercial Sex Workers stratified by Virologic Status

Variable	Virologic Suppression (N=120)	Virologic non-suppression (N=20)	Odds Ratio (95% C.I.)	P-value
**Age**, median (IQR)	27 (25 –32)	30 (27 –32)	0.97 (0.90 –1.04)	0.35
**History of Tuberculosis**				
No	116 (99.1%)	17 (85.0%)	1	0.01
Yes	1 (0.9%)	3 (15.0%)	0.05 (0.01–0.5)	
**History of S.T.I.s**				
No	88 (75.9%)	13 (65.0%)	1	
Yes	28 (24.1%)	7 (35.0%)	0.59	0.31
**Baseline CD4 categorized**				
CD4 count < 500 cells/µL	72 (61.0%)	15 (75.0%)	1	0.24
CD4 count > 500 cells/µL	46 (39.0%)	5 (25.0%)	1.92 (0.65 – 5.63)	
**Baseline Hemoglobin** (g/dl), median (IQR)	12.05 (11.1– 13.2)	12.8 (11.7 . 13.4)	0.81 (0.47 –1.39)	0.45
**Baseline ART Regimen**, Zidovudine-based	16 (13.3%)	4 (21.1%)	1	0.38
regimen	104 (86.7%)	15 (78.9%)	1.73(0.51–5.88)	
Tenofovir-based regimen				
**Time duration from** **enrolment in care to start of** **ART**	58 (48.7%)	1 (5.0%)	1	
Delayed start (≥3 weeks)	43 (36.1%)	13 (65.0%)	0.57 (0.01–0.45)	<0.02
Immediate start (<2 weeks)	18 (15.2%)	6 (30.0%)	0.52 (0.01–0.46)	
Transfer-in while on ART				
**Adherence to ART**				
Good (>95%)	93 (81.6%)	11 (61.1%)	1	
Fair (85–94%)	14 (12.3%)	5 (27.8%)	0.33 (0.1 –1.1)	0.15
Poor (<84%)	7 (6.1%)	2 (11.1%)	0.41 (0.08 –2.25)	
**Care Entry Point**				
Out-Patient Department	45 (61.6%)	3 (21.4%)	1	
Community outreach	17 (23.3%)	8 (57.2%)	0.14 (0.03–0.60)	0.03
Antenatal clinic	2 (2.8%)	0		
Transfer-in	9 (12.3%)	3 (21.4%)	0.2 (0.03–1.15)	

P-value - Overall wald test p-value from logistic regression, ART - Antiretroviral therapy, S.T.I - Sexually transmitted infection. Virologic Suppression = viral load <1000 copies/ml.

Missing; History of tuberculosis 3/140 (2.1%), History of S.T.I.s 4/140 (2.9%), Baseline CD4 cell count 2/140 (1.4%), Baseline hemoglobin 97/140 (69.3%), Baseline ART Regimen 1/140 (0.7%), time to ART Initiation 1/140 (0.7%), Adherence to ART 8/140 (5.7%), Care Entry Point 55/140 (39.3%).

**Table 3:** Baseline Characteristics of Female General Population stratified by Virologic Status

Variable	Virologic Suppression N=163	Virologic non-suppression N=19	Odds Ratio (95% C.I.)	P-value
**Age**, median (IQR)	31 (26 – 37)	32 (25 – 37)	1.0 (0.96 – 1.06)	0.78
**History of Tuberculosis**				
No	155 (96.3%)	17 (89.5%)	1	0.19
Yes	6 (3.7%)	2 (10.5%)	0.33 (0.06 – 1.75)	
**History of S.T.I**				
Yes	6 (4.2%)	0	1	
No	137 (95.8%)	16 (100%)	-	-
**Baseline CD4 categorized**				
CD4 count < 500 cells/µL	123 (75.5%)	17 (89.5%)	1	0.19
CD4 count > 500 cells/µL	40 (24.5%)	2 (10.5%)	2.76 (0.6 – 12.5)	
**Baseline Hemoglobin(g/dl)** median (IQR),	12.1 (11.0 – 13.7)	11.9 (11.6 – 13.6)	0.97 (0.62 – 1.51)	0.87
**Baseline ART Regimen**				
Zidovudine-based regimen	49 (30.1%)	7 (36.8%)	1	0.55
Tenofovir-based regimen	114 (69.9%)	12 (63.2%)	1.36 (0.5 – 3.7)	
**Duration from enrolment** **in care to start of** ART (months) Delayed start (.3 weeks)	90 (55.9%) 49 (30.4%)	10 (52.6%) 5 (26.3%)	1 1.09 (0.35 – 3.37)	0.69
Immediate start (<2 weeks)	22 (13.7%)	4 (21.1%)	0.61 (0.18 –2.13)	
Transfer-in while on ART				
**Adherence to ART**				
Good (>95%)	152 (93.8%)	15 (83.3%)	1	0.26
Fair (85–94%)	8 (5.0%)	2 (11.1%)	0.39 (0.08 –2.03)	
Poor (<84%)	2 (1.2%)	1 (5.6%)	0.20 (0.02 – 2.31)	
**Care Entry Point**				
Out-Patient Department	89 (73.0%)	12 (92.3%)	1	
Community outreach	1 (0.8%)	0		
Antenatal Clinic	28 (23.0%)	1 (7.7%)	3.64 (0.45 –29.28)	0.22
Transfer-in	3 (2.4%)	0		
TB Clinic	1 (0.8%)	0		

P-value - Overall wald test p-value from logistic regression, ART – Antiretroviral therapy, S.T.I – Sexually transmitted infection. Virologic Suppression = viral load <1000 copies.ml. Missing; History of tuberculosis 2/182 (1.1%), History of S.T.I.s 29/182 (15.9%), Baseline hemoglobin 139/182 (76.4%), time to ART Initiation 3/182 (1.6%), Adherence to ART 2/182 (1.1%), Care Entry Point 52/182 (28.6%).

CSW who had a positive history of tuberculosis had 0.05 the Odds (95% Confidence Interval 0.01 – 0.5, P = 0.01) of achieving virologic suppression compared to those who had a negative history of tuberculosis. Similarly, GPs who had a positive history of tuberculosis had 0.33 Odds (95% confidence interval 0.06 – 1.75) of achieving virologic suppression but the association was statistically insignificant (P = 0.19).

CSW who initiated ART <2 weeks from enrolment in care had 0.57 the Odds (95% confidence intervals 0.01 – 0.45) of achieving virologic suppression compared to those who initiated ART initiation ≥3 weeks. Similarly, CSW who transferred from other treatment centres to the study centres (Transfer-In) had 0.52 Odds (95% confidence intervals 0.01 – 0.46) of achieving virologic suppression compared to those who delayed ART initiation. Both results were statistically significant, P = 0.02.

In the female GP, duration to ART initiation from time of enrolment in care was not associated with virologic suppression. Those who initiated ART < 2 weeks had 1.09 odds (95% confidence intervals 0.35 – 3.37, P = 0.69) of achieving virologic suppression compared to those who delayed ART initiation ≥ 3 weeks.

In the multivariate analysis (Table 4) based on a causal pathway model (Adherence was not adjusted for), factors associated with virologic suppression in the CSWs were history of tuberculosis and time to ART initiation. CSW who had a positive history of tuberculosis had 0.05 the odds (95% confidence interval 0.01 – 0.96, P = 0.046) of achieving virologic suppression compared to those with a negative history of tuberculosis.

**Table 4:** Logistic Regression Model of Virologic Suppression in the Commercial Sex Workers

Variables	Unadjusted Odds (95% C.I)	P-value	Adjusted Odds (95% C.I)	P-value
History of Tuberculosis				
No	1	0.01	1	0.046
Yes	0.05 (0.01–0.50)		0.09 (0.01 – 0.96)	

**History of STD**				
**No**	1	0.31	1	0.92
**Yes**	0.59 (0.21–1.63)		0.94 (0.29 – 3.02)	

**Time duration from** **enrolment to start of ART**				
Delayed start (≥3 weeks)	1		1	
Immediate start (<2 weeks)	0.57 (0.01–0.45)	0.008	0.07 (0.01 – 0.55)	0.01
Transfer in	0.52 (0.01–0.46)	0.007	0.07 (0.01 – 0.65)	0.02

CSW who initiated ART <2 weeks from time to enrolment in care had 0.07 the odds (95% confidence intervals 0.01 – 0.55, P = 0.01) of achieving virologic suppression compared to those who delayed ART initiation ≥3 weeks. Similarly, transfer-in CSW had 0.07 the odds (95% confidence intervals 0.01 – 0.65, P = 0.02) of achieving virologic suppression compared to those who delayed ART initiation by ≥ 3 weeks. In the female GPs, no variable was associated with virologic suppression at multivariate analysis.

## Discussion

### Virologic Suppression

Virologic suppression (Viral load <1000 copies/ml) after 6 months (24 weeks) of ART was similar in the female general population (89.6%) and the sex workers (85.7%). These findings are similar to other studies in conducted in the Ugandan general HIV population by Bulage, Castelnuovo and Grabowski where virologic suppression (viral load <1000 copies/ml) was 89%, 93% and 94% respectively^6^,^19^,^20^. Similarly, CSW in our study achieved high levels of virologic suppression as a similar study involving Ugandan CSW where virologic suppression was 93%^21^. Other African studies done in Zimbabwe and Malawi showed relatively high levels of virologic suppression in CSW at 77.8% and 86% respectively^12^,^22^. When considering virologic suppression stratified to the lowest undetectable level (<75 copies/ml) versus any detectable level, virologic suppression was equally similar. These findings suggest that HIV key population groups who initiate and remain on ART achieve good virologic outcomes as the general HIV population.

However, the overall level of virologic suppression in the CSW was low at 55% when considering virologic suppression as viral load <1000 copies/ml, and 51.8% when considering virologic suppression = undetected viral load. This low overall virologic suppression is similar to data reported from studies done in Zimbabwe and Malawi, where overall virologic suppression among the CSWs was 49.5% and 45% respectively^12^,^22^. These observations fall short of the UNAIDS target of achieving 90% virologic suppression10 emphasizing the need to improve treatment outcomes for HIV key populations.

### Factors associated with HIV Virologic Suppression

In our study, we observed that the majority of the CSWs initiated ART <2 weeks from time of enrolment in care, in line with the WHO 2014 guidelines where key population groups were immediately started on ART regardless of their immune status or WHO stage^1^. In comparison, most female GP patients were initiated on ART beyond 2 weeks from the time of enrolment in care. This was prior to the adoption of the “test and treat” strategy that was implemented in 2016 16.

However, we observed that the CSW who immediately initiated ART < 2 weeks from time of enrolment in care were less likely to achieve virologic suppression with an adjusted odds ratio of 0.07 compared to those who delayed ART initiation ≥3 weeks. Possible explanations to this observation could be that the CSW who immediately started ART may not have been adequately counselled and prepared for ART initiation, as reflected by the high levels of suboptimal adherence to ART and the high rate of attrition at 71% and 23% respectively. We cannot confidently conclude that delaying ART initiation predicts better virologic outcomes in key populations. Multiple factors could confound and explain this observation such as the several barriers to treatment access that weren't measured and adjusted for in this study. Sex workers report several barriers to treatment access and retention in care such as stigma, physical violence, discrimination by health workers, criminalization and imprisonment^13^,^14^. Furthermore, current evidence proves that early initiation of ART has benefits in reducing HIV mortality and improving treatment outcomes^23^. Larger prospective studies are still needed to investigate the factors that predict virologic suppression in key populations in the local context.

Tuberculosis predicted non-virologic suppression among the CSWs in our study. Those with a positive history of tuberculosis were less likely to achieve virologic suppression, with an adjusted odds ratio of 0.05. This tends to agree with data from a South African cohort where tuberculosis had a negative association with virologic suppression in a younger HIV population^24^. However, a systematic review 49,000 HIV positive adults showed no association between tuberculosis treatment and virologic suppression, with an odds ratio of 0.97 95% confidence intervals 0.92 – 1.03^25^. Given the heterogeneity of data between tuberculosis and virologic suppression in different population groups, more research is warranted to derive substantive conclusions.

### Retention in care in key populations

In this study, 23% of the CSWs were lost-to-follow-up after 6 months of follow-up. This finding agrees with a study in Kenya where the level of attrition in CSW was reported at 21% 26 although a study in Burkina Faso reported much lower attrition rates at 13%^27^. Although these studies had longer follow up periods at one and three years respectively, these high drop-out rates emphasize the need to strengthen retention of key populations in care.

We were unable to measure retention in care in the general population in our study given the fact that GPs were selected on the basis of having viral load result which inadvertently affected retention in care. However, other studies involving the general HIV population show relatively high retention in care rates at 97% and 95% in a Ugandan and South African cohort^28^,^29^. This suggests that retention rates in the general HIV population might be higher than in key populations.

The low retention in care rate among key populations such as sex workers is probably due to certain intrinsic characteristics such as their high mobility or unique barriers to access to treatment. A study in Zimbabwe showed that CSW frequently changed their living addresses, suggesting high mobility within the population^12^. Factors such as stigma, social discrimination, mistreatment or rejection by health workers, violation of privacy and fear of disclosure have been documented as barriers hindering sex workers' access to treatment and retention in care^30^,^31^. This reflects the uniqueness and vulnerability of the key populations and the need to remove barriers that hinder their access to treatment.

## Strengths, Limitations and Recommendations

Our study compares the virologic suppression of key populations within the same study setting as the general population, informing of the shortfalls in their care cascade in relation to the UNAIDS 90-90-90 targets. Despite achieving comparable virologic suppression rates, key populations are prone to high attrition and may require unique interventions to improve their treatment outcomes.

However, we were limited in our inability to measure retention in care for the female general population and follow up the lost patients. We recommend conducting large prospective cohort studies to fully understand the dynamics of the HIV care cascade in key populations.

## Conclusion

After six months from ART initiation, virologic suppression is similar between female sex workers and the female general population. However, the low overall virologic suppression and high attrition rates could jeopardize the success of the 90-90-90 targets unless population-specific interventions are implemented to improve retention and treatment outcomes.

## Figures and Tables

**Figure 1:**
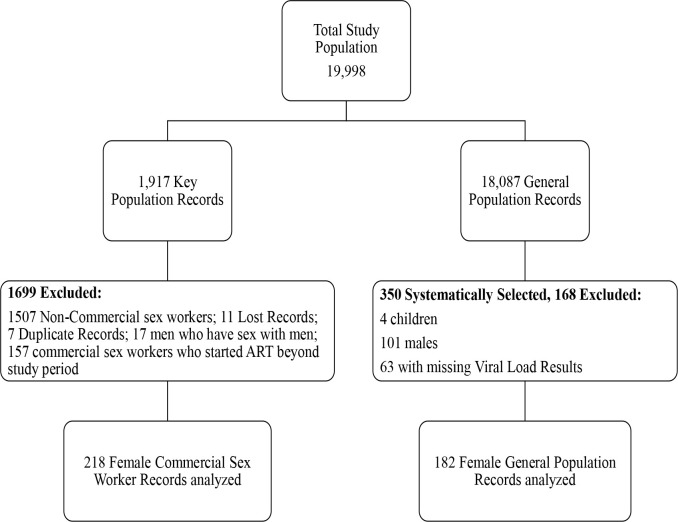
Study Flow Diagram
